# Supplementary material for: Genome-Wide Association Study for Udder Conformation Traits in Chinese Holstein Cattle
Source: Animals (Basel). 2022 Sep 22;12(19):2542. doi: 10.3390/ani12192542 (PMC9559277; doi:10.3390/ani12192542)

Figure S1. The distribution of the SNP information on 29 chromosomes within 1 Mb window size.

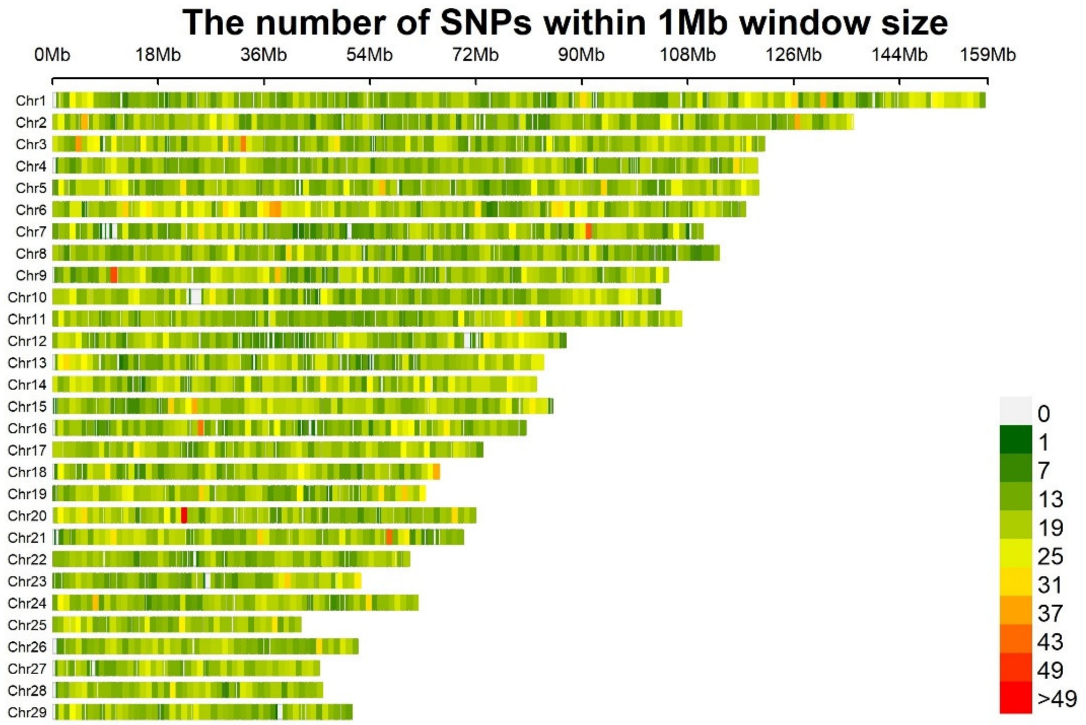

Figure S2. The distribution and correlation of the phenotypes among the udder structure traits in Chinese Holstein cattle.

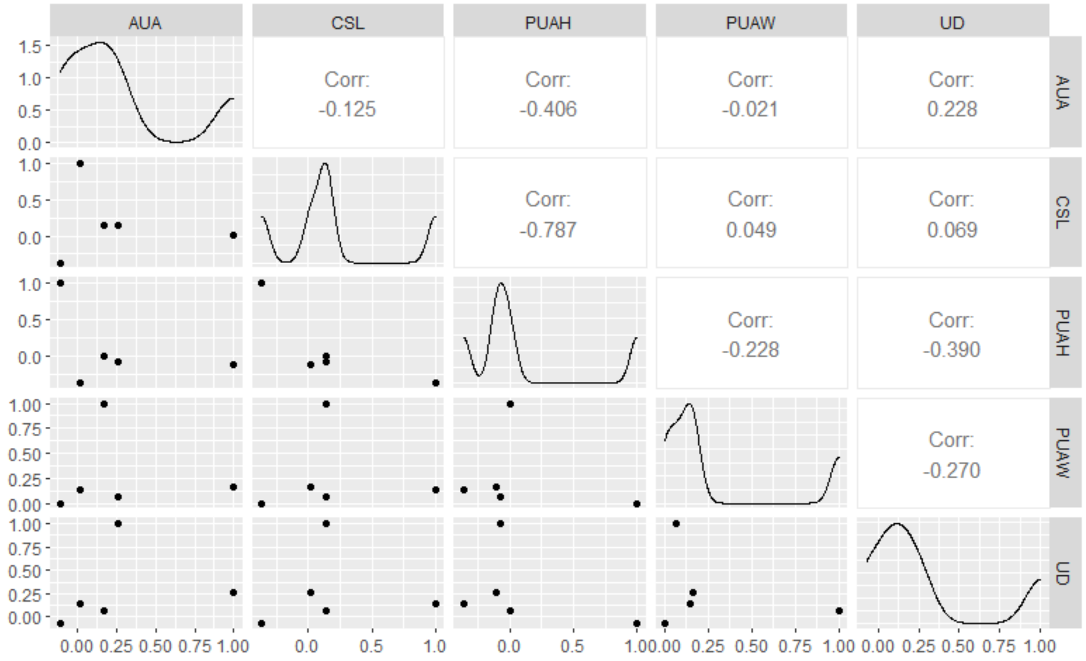

Figure S3. The genotypic correlation among the udder structure traits in Chinese Holstein Cattle.

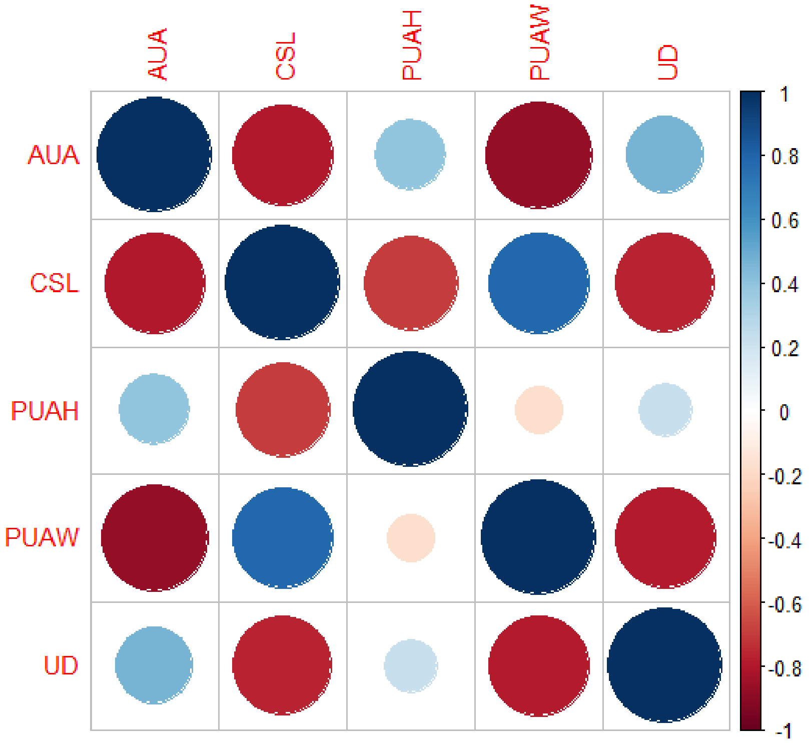

Figure S4. Kyoto encyclopedia of genes and genomes (KEGG) pathways analysis for udder structure conformation traits.

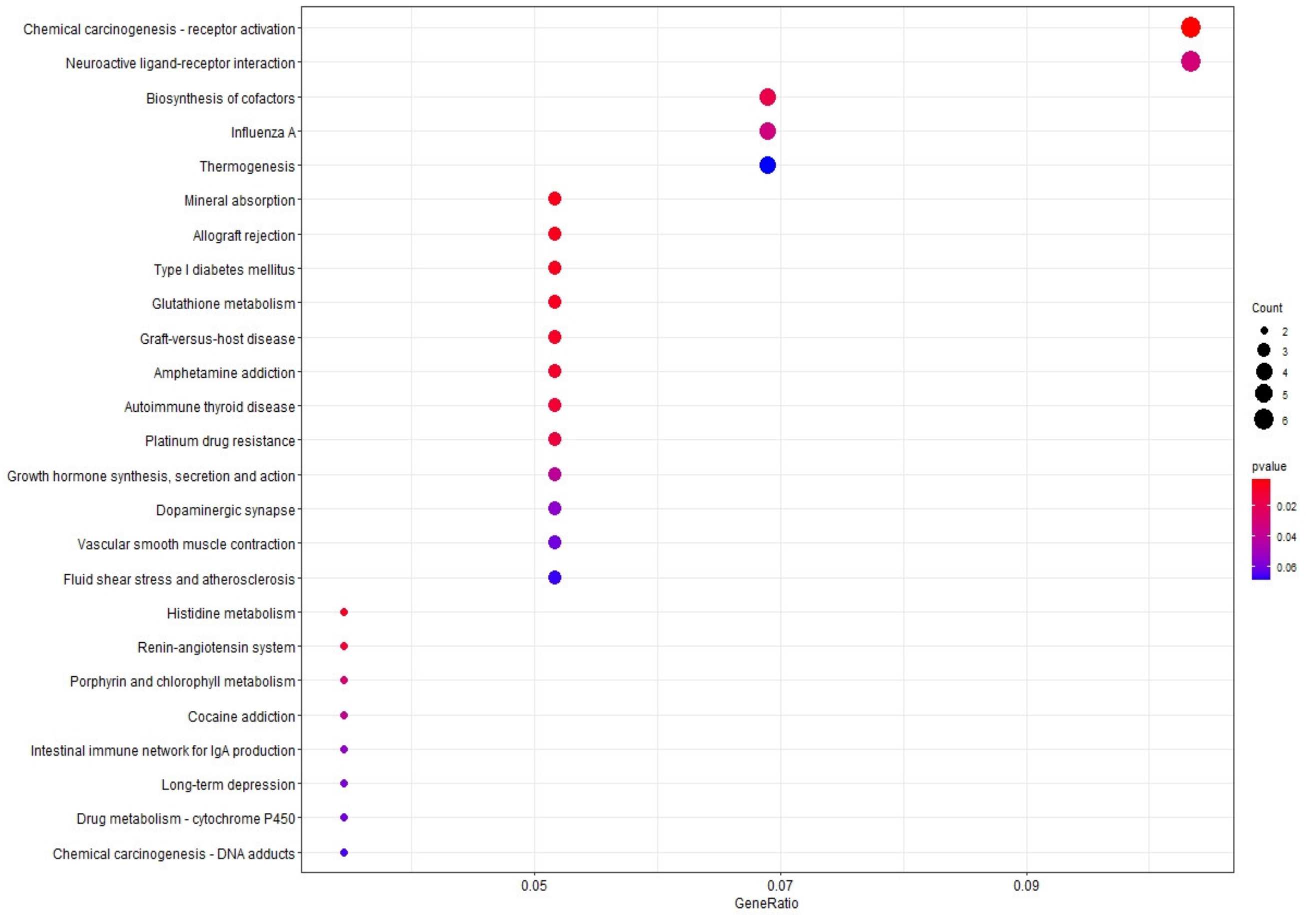

Supplement: Supplementary file 1 [file animals-12-02542-s001.zip › Suplimentary Figures.pdf]
